# Exudative retinal detachment and hypertensive choroidopathy in a patient with suspected HELLP syndrome: a case report

**DOI:** 10.1186/s12886-025-04176-8

**Published:** 2025-07-01

**Authors:** Muzi Li, Jinfeng Qu

**Affiliations:** Department of Ophthalmology, Peking University People’s Hospital, Beijing, China

**Keywords:** Severe preeclampsia, HELLP syndrome, Malignant hypertension, Hypertensive retinopathy, Visual impairment

## Abstract

**Background:**

The HELLP syndrome is a serious complication in pregnancy, characterized by haemolysis, elevated liver enzymes and low platelet count. We report a patient who was unaware of pregnancy presenting initially with bilateral visual loss and exudative retinal detachment and was diagnosed with HELLP syndrome during work-up.

**Case presentation:**

A female patient, unaware of her pregnancy, presented with painless, severe bilateral vision loss and exudative retinal detachment. Multimodal imaging of her fundus revealed hypertensive choroidopathy and retinopathy. During systemic work-up, she was found to be 26 weeks pregnant and was diagnosed with HELLP syndrome based on laboratory abnormalities. Her vision fully recovered within 4 months following urgent pregnancy termination and blood pressure control.

**Conclusions:**

This case highlights the importance of obtaining a thorough medical history and considering pregnancy-related complications in the differential diagnosis for reproductive-age women with acute vision loss. Prompt diagnosis and treatment of this condition are essential for the recovery of patients’ visual outcome.

## Introduction

Visual changes during pregnancy and the perinatal period can be physiological, but they may also indicate serious pathological conditions such as preeclampsia [1, 2]. Preeclampsia is a major global contributor to maternal morbidity and mortality [3, 4], typically occurring after 20 weeks of gestation [4]. It is defined by the onset of new hypertension accompanied by at least one of the following symptoms: proteinuria, thrombocytopenia, renal dysfunction, liver dysfunction, pulmonary edema, or refractory headache [3, 4]. Preeclampsia can progress to life-threatening complications including eclampsia, HELLP syndrome (characterized by hemolysis, elevated liver enzyme and low platelet count), stroke, or even maternal death. Affected individuals may also suffer long-term damage to organs such as the kidneys, liver, heart, and eyes [3].

Preeclampsia can impact multiple components of the visual system, including the retina, optic nerve, and occipital cortex [1, 5]. Severe preeclampsia may result in optic disc edema due to elevated intracranial pressure [1]. Both severe preeclampsia and eclampsia are associated with a heightened risk of hypertensive retinopathy and choroidopathy, central retinal vein occlusion, strokes, and various visual disturbances [1, 6, 7].

We present the case of a female patient with preeclampsia and suspect HELLP syndrome who developed exudative retinal detachment and hypertensive choroidopathy.

## Case report

**Fig. 1:**
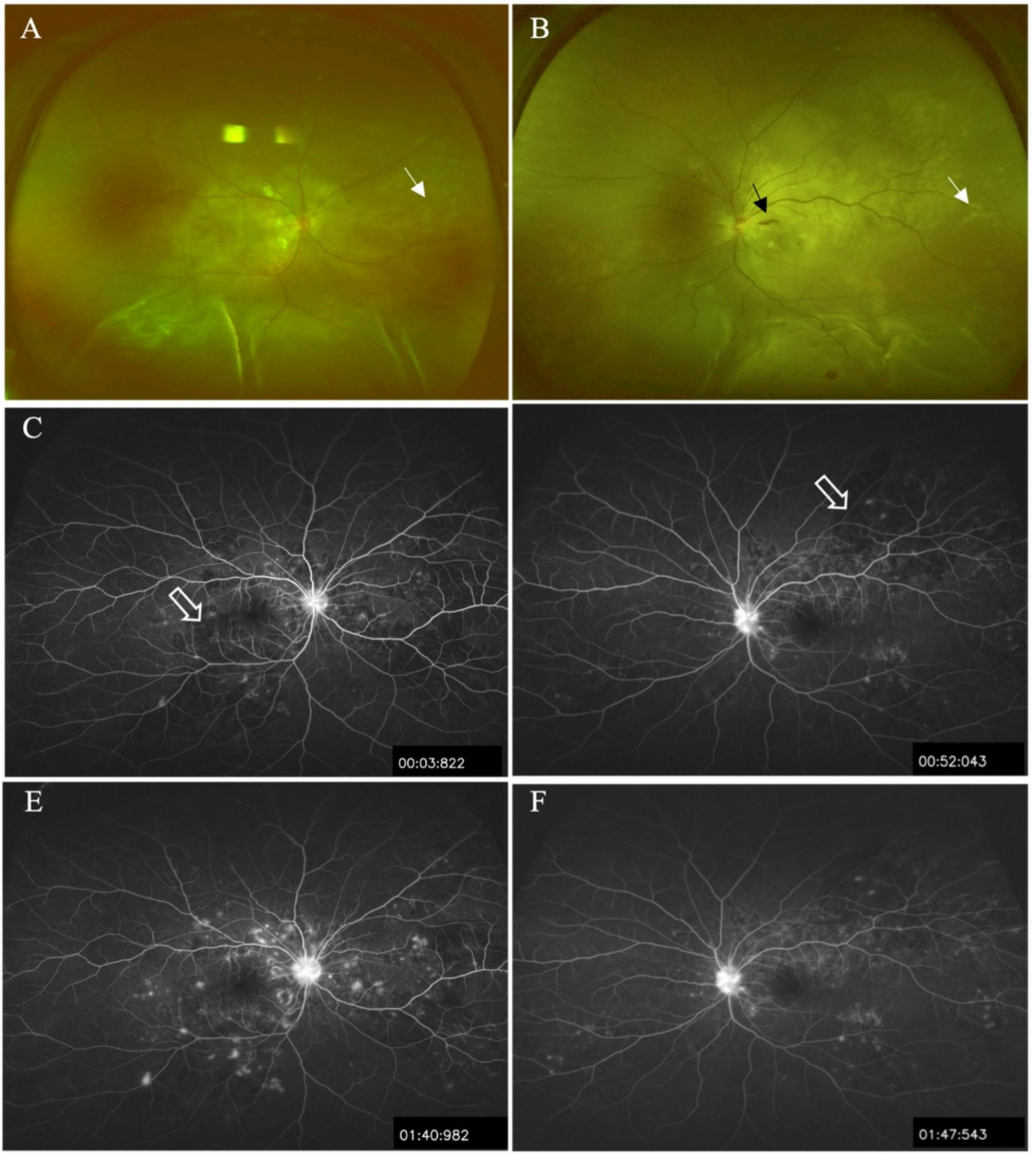
Ultra-wide field fundus photography of both eyes **A** and **B** revealed bilateral retinal detachment in the inferior quadrants accompanied by mild disc edema, arterial narrowing, moderate venous dilation, mild splinter retinal hemorrhage (black arrow), and Elschnig spots (white arrow) in the posterior pole and mid-peripheral. Fundus Fluorescein Angiography (FFA) showed patchy delayed choroidal filling (blank arrow) in the posterior pole and in nasal quadrant of the right eye, as well as the supertemporal quadrant of the left eye. Multiple hyperfluorescent dots were observed in the early phase **C** and **D**, with late-phase leakage **E** and **F** in the posterior pole and mid-peripheral regions of both eyes

 A 39-year-old Chinese female patient presented to the ophthalmology department on April 18th 2024 with visual impairment. She reported a sudden, painless severe loss of vision in both eyes which began 8 days earlier. Her best corrected visual acuity (BCVA) was light perception (LP) in both eyes. Her intraocular pressure was 18mmHg in the right eye and 16mmHg in the left eye. The ocular motility exam was normal. No relative afferent pupillary defects were identified. She had mild conjunctival hyperemia and edema in both eyes. The slit lamp examinations were unremarkable for both eyes. Fundus examination revealed exudative retinal detachment (ERD) in the inferior quadrants of both eyes, accompanied by mild disc edema, arterial narrowing, moderate venous dilation, mild splinter retinal haemorrhage, cotton wool spots, and deep yellow lesions known as Elschnig spots in the posterior pole and mid-periphery (Fig. 1A and B).

**Fig. 2:**
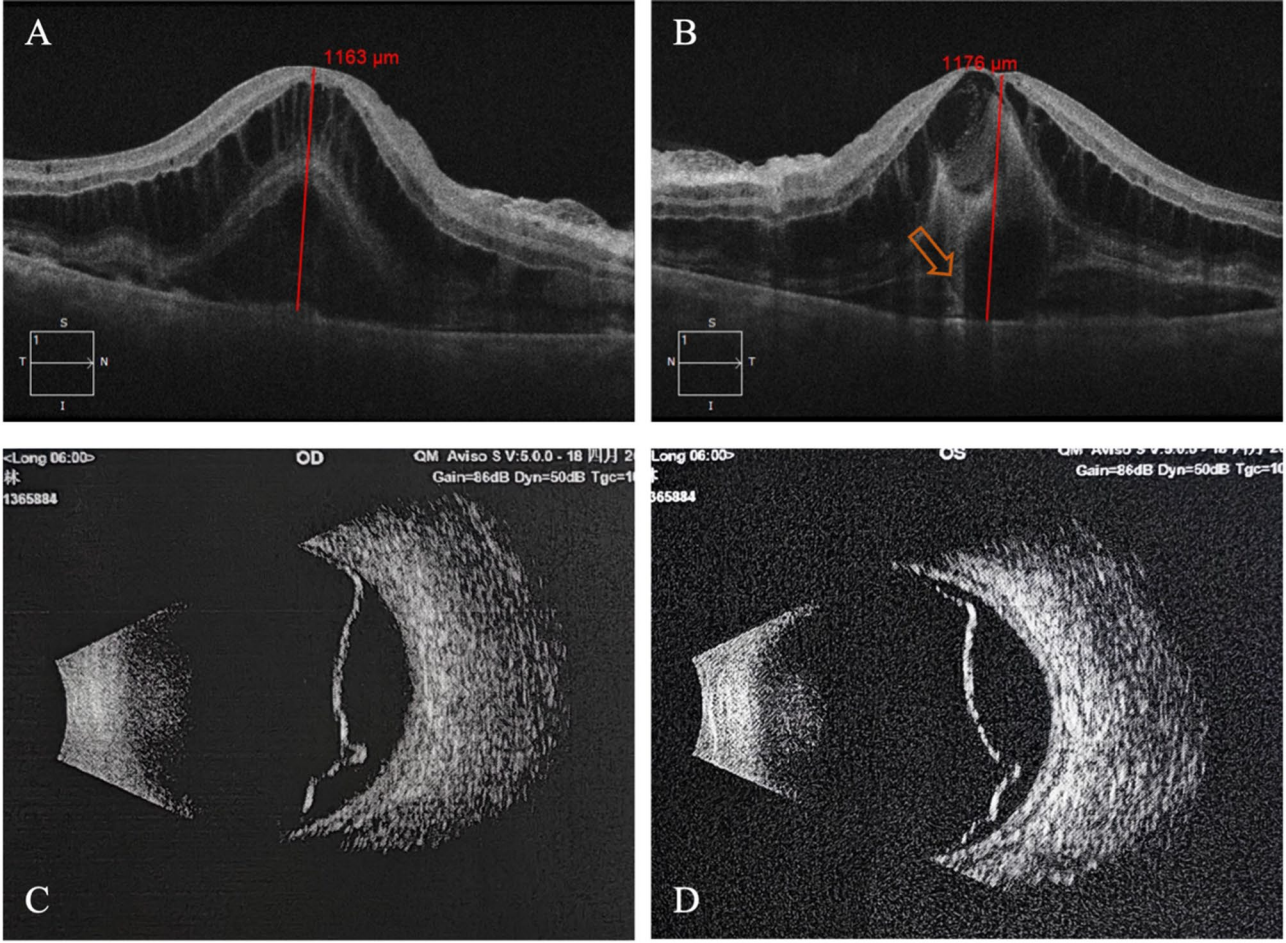
Spectral Domain optical coherence tomography (SD-OCT) B-scan **A** and **B** and Ultra-sound B scan images of both eyes **C** and **D** in this patient at the first visit. The red blank arrow indicates bacillary layer detachment (BALAD) sign on OCT

Fundus Fluorescein Angiography (FFA) revealed patchy delayed choroidal filling (blank arrow) in the posterior pole and in nasal quadrant of the right eye, as well as the supertemporal quadrant of the left eye. Multiple hyperfluorescent dots were observed in the early phase, with late-phase leakage in the posterior pole and mid-peripheral regions of both eyes (Fig. 1C and F). Spectral Domain optical coherence tomography (SD-OCT) B-scan through the fovea demonstrated ERD involving the fovea. The choroid was thickened with the outer boundary invisible in the scanned area in both eyes. SD-OCT also revealed prominent intraretinal fluid (IRF) in the outer nuclear layer (ONL) and subretinal fluid (SRF) in both eyes. Manually measured central retinal thickness, using the built-in calliper tool, was 1163 μm in the right eye and 1176 μm in the left eye. A bacillary layer detachment (BALAD) sign was present at the foveal center of the left eye (Fig. 2A and B). B-scan ultrasonography confirmed the presence of exudative retinal detachment in both eyes.

These ocular findings were suggestive of hypertensive retinopathy and choroidopathy. Her blood pressure, measured in the ophthalmology department, was 183/136 mmHg. A thorough medical history was subsequently obtained. She reported a history of irregular menstruation, occurring every 60–90 days with each cycle lasting for 4–5 days with a small amount of menstrual flow without dysmenorrhea. Her last menstrual period was reportedly one week prior.

She was diagnosed with hypertension over one month earlier at a local hospital and was prescribed Telmisartan (one tablet once daily) and Nifedipine (one tablet twice daily). However, her blood pressure remained poorly controlled, fluctuating between 160–180/100–110 mmHg, but she did not attend follow-up appointments.

One week prior to her presentation in our hospital, she began experiencing blurred vision in both eyes, abdominal distension, chest tightness, and shortness of breath. Three days before admission, she developed nausea and vomiting, occurring 4–5 times, prompting her to visit the emergency department. There, a pelvic ultrasound unexpectedly revealed an intrauterine pregnancy. Her lab results revealed a decreased platelet count (66 × 10*9/L) and elevated levels of AST, ALT and LDH. Obstetric ultrasound estimated a gestational age at 26 + weeks.

Her diagnose was revised to chronic hypertension complicated by severe preeclampsia and suspected HELLP syndrome. She was treated with intravenous magnesium sulfate, urapidil, dexamethasone and hepatoprotective agents. After a multidisciplinary consultation, the severity of her condition was explained to her, and she consented to undergo mid-trimester pregnancy termination.

**Fig. 3:**
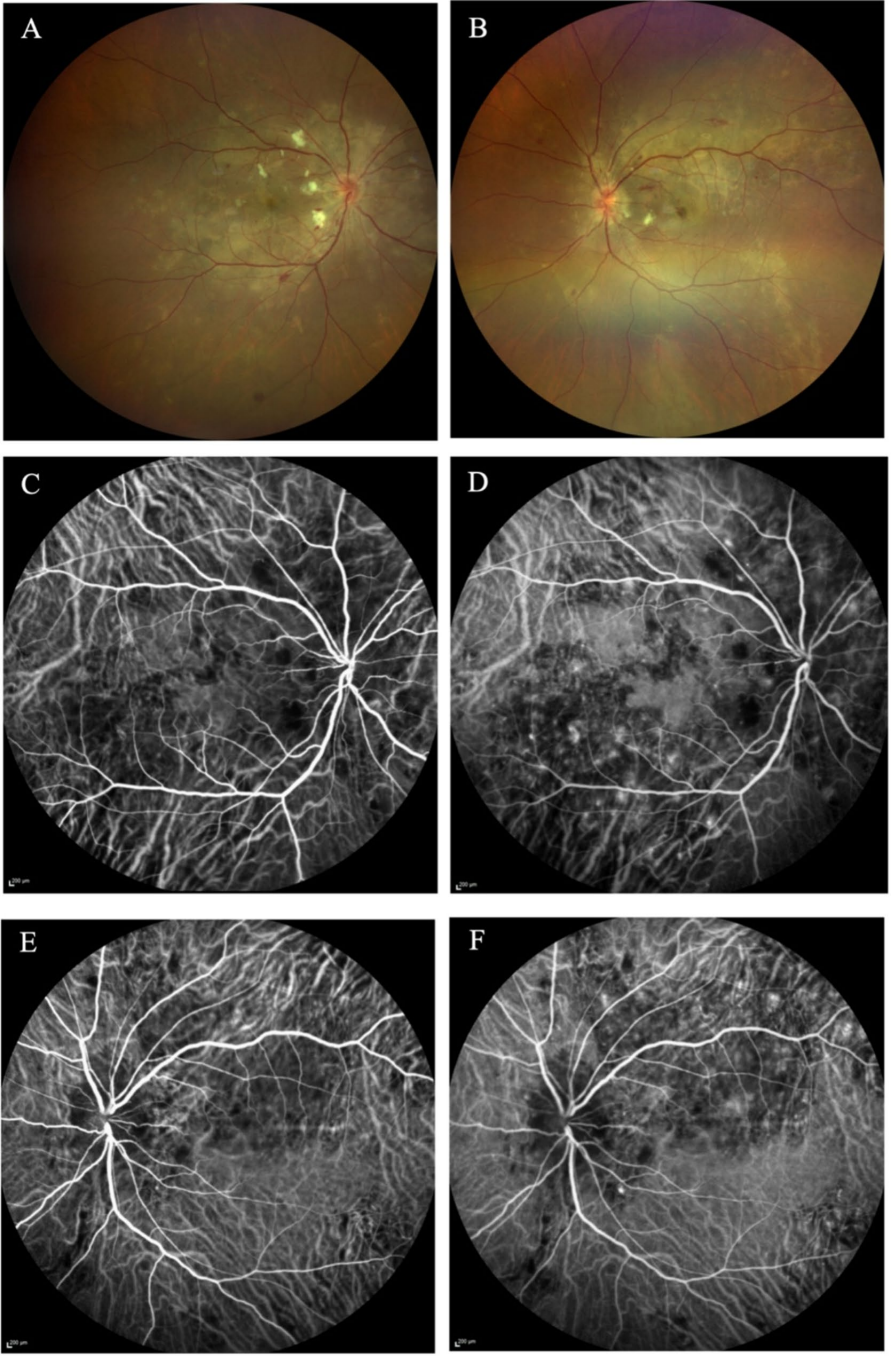
Fundus photography **A** and **B** showed resolution of exudative retinal detachment and improvement of disc edema, arterial narrowing and Elschnig spots in both eyes. ICGA **C** and **F** showed prominent choroidal ischemia around the optic disc in bath eyes, infratemporal quadrant of the right eye, superior temporal and inferior quadrant of the left eye

Five days after the induced labor, her BCVA went back to 0.4 in the right eye and 0.5 in the left eye. She was prescribed oral nifedipine and valsartan, and her blood pressure decreased to 150/100mmHg. The fundus examination revealed resolution of exudative retinal detachment and disc edema. Elschnig spots appeared less deep in both eyes (Fig. 3A and B). Indocyanine green angiography (ICGA) showed prominent bilateral choroidal ischemia (Fig. 3C and F). She received a single retrobulbar injection of anisodamine and was prescribed oral mecobalamin and ginkgo biloba extract for one month.

**Fig. 4:**
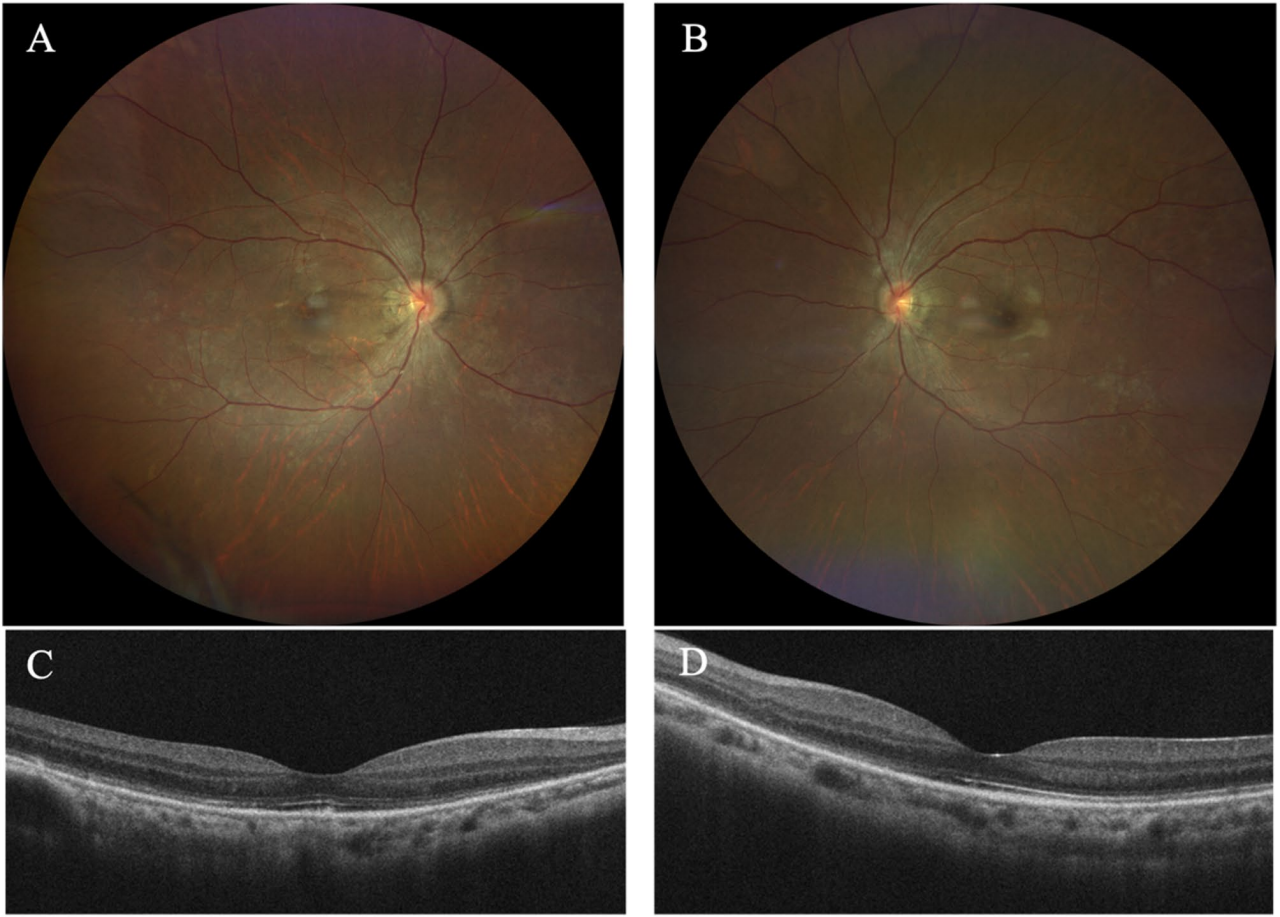
Fundus photography at four months visit **A** and **B** showed Elschnig spots and cotton wool spots disappeared but the retinal artery narrowing still remained. SD-OCT **C** and **D**) showed totally resolve of IRF and SRF, choroidal thickness went back to normal and mild RPE proliferation in both eyes

One month after the initial visit, her BCVA improved to 0.6 in both eyes and her blood pressure was well controlled. Fundoscopy revealed Elschnig spots almost disappeared and cotton wool spots had resolved. Four months after the initial visit, her BCVA further improved to 0.8 in both eyes. Fundoscopy showed complete resolution of cotton wool spots, although retinal artery narrowing persisted (Fig. 4A and B). SD-OCT showed complete resolution of IRF and SRF, and choroidal thickness had returned to normal. However, the disorganization of external limit membrane (ELM) and ellipsoid zone (EZ) was still present nasal to the foveal center, along with mild retinal pigment epithelium (RPE) proliferation in both eyes. (Figure 4C and D).

## Discussion

This case report presents a woman with bilateral severe visual loss and ERD who was unaware of pregnancy initially and was diagnosed with HELLP syndrome during work-up. Her history of irregular menstruation contributed to her unawareness of a missed period. Her obesity and possible fetal growth restriction which is commonly associated with preeclampsia may have led to mild abdominal prominence and further obscured the recognition of pregnancy by herself and her family. Moreover, the minor decidual bleeding during pregnancy may also have been misinterpreted as “menstruation”, which made the diagnosis of pregnancy-related complications more challenging. This case highlights the importance of taking a thorough medical history and considering pregnancy-related complications in the differential diagnosis of acute vision loss in women of reproductive age.

HELLP syndrome is an extreme manifestation of severe preeclampsia. A series of recent studies have revealed the effects of HELLP syndrome on the eyes, particularly with retinal and choroidal lesions. Ocular complications of HELLP syndrome also include ERD which has a higher incidence among patients with severe preeclampsia [8–11].

Zamora et al. described a case of a patient presenting with HELLP syndrome and ERD after a cesarean section. These ocular lesions are believed to result from hypertensive retinopathy and choroidal ischemia. The combination of hypertension, microvascular disease, hypercoagulable state, and hypoalbuminemia is thought to play a key role in the development of ERD [8]. Additionally, Bos et al. shows that retinal detachment is a rare but recognized complication of preeclampsia and eclampsia, which can occur in either prepartum or postpartum. Retinal and choroidal vascular injury is considered the pathological basis for its occurrence, and fluorescein angiography observations support the hypothesis that retinal detachment is secondary to choroidal ischemia [9].Our case provides high quality and classic multimodal imaging of a HELLP syndrome with ERD and hypertensive choroidopathy.

Hypertensive chorioretinopathy is a well-recognized complication of preeclampsia, its detailed mechanisms, and hemodynamic changes are continually being investigated [11, 12]. Vascular regulation in the retina and choroid was believed to play an important role in the process of hypertensive retinopathy and choroidopathy [13]. Patients with hypertensive chorioretinopathy often exhibit increased choroidal blood flow and choroidal thickness, suggesting that choroidal hyperperfusion significantly contributes to the disease progression [14]. Giuseppe et al. and Giulio et al. further explored the relationship between choroidal thickness and renal hemodynamics, finding that changes in choroidal thickness are related to systemic arterial damage. This finding suggested that vascular elasticity changes in both eye and kidney may be mediated by oxidative stress and endothelial dysfunction [15, 16]. Choriocapillaris non-perfusion can weaken the pump function of RPE, leading to ERD and subsequent outer retinal damage. Our case demonstrates choroidal ischemia plays an important role in the development of ERD. Despite a reduction in blood pressure to 150/100 mmHg, signs of choroidal ischemia persisted in our patient. Unfortunately, her ICGA images at 4 months follow-up was not available to further assess the progression of choroidal changes.

Regarding the treatment of HELLP syndrome, Schiffrin et al. studied the effects of the angiotensin receptor antagonist Losartan on vascular structure and endothelial function in hypertensive patients, showing that Losartan can reverse structural changes in resistant arteries and improve endothelial dysfunction, whereas the β-blocker atenolol does not have these effects [17]. Comparative studies between Perindopril Argino and Amlodipine in hypertensive patients also showed that Perindopril Argino significantly increased choroidal thickness, whereas Amlodipine does not produce notable changes [18].

Our case highlights that prompt pregnancy termination and rigorous blood pressure control are critical for achieving full visual recovery in HELLP syndrome. Additionally, we explored adjunctive therapies targeting microcirculatory improvement and neuroprotection in our case. Anisodamine, an M-type cholinergic receptor antagonist, was administered via retrobulbar injection to alleviate choroidal ischemia. This agent has been shown to relieve vasospasm, enhance microcirculation, and inhibit vascular permeability [19] and is clinically used in central retinal artery occlusion and ischemic optic neuropathy. Ginkgo biloba extract, which has demonstrated efficacy in managing peripheral vascular disorders and cerebral circulatory insufficiency, was used to reduce retinal vascular permeability and macular edema [20]. Mecobalamin, a well-established neuroprotective agent [21, 22] was administered to minimize retinal neural damage. However, the efficacy and role of these adjunctive therapies in hypertensive chorioretinopathy require further investigation.

## Conclusions

HELLP syndrome is closely related to a range of pathological changes in the retina and choroid, which not only affect the patient’s vision but may also serve as indicators of the severity of systemic vascular injury. Although current research has shed light on several important aspects, many underlying mechanisms still require further exploration. Effective management of HELLP syndrome requires not only prompt pregnancy termination to reverse ocular manifestations and prevent permanent visual impairment but also a comprehensive understanding of the disease’s etiology and progression. This approach is essential to guide the development of more targeted and effective treatment strategies.

## Data Availability

No datasets were generated or analysed during the current study.

## References

[1] Gilbert AL, Prasad S, Mallery RM. Neuro-Ophthalmic disorders in pregnancy. Neurol Clin. 2019;37:85–102. 30470277 10.1016/j.ncl.2018.09.001

[2] He X, Ji Y, Yu M, Tong Y. Chorioretinal Alterations Induced by Preeclampsia. J. Ophthalmol. 2021;8847001. 10.1155/2021/8847001 PMC7969093 33777446

[3] Chappell LC, Cluver CA, Kingdom J, Tong S. Pre-eclampsia. Lancet. 2021;398:341–54. 34051884 10.1016/S0140-6736(20)32335-7

[4] Gestational Hypertension and Preeclampsia. ACOG practice bulletin, number 222. Obstet Gynecol. 2020;135:e237–60. 32443079 10.1097/AOG.0000000000003891

[5] Digre KB. Neuro-Ophthalmology and pregnancy: what does a Neuro-Ophthalmologist need to know? J Neuroophthalmol. 2011;31:381–7. 22089502 10.1097/WNO.0b013e31823920cb

[6] Soma-Pillay P, Pillay R, Wong T, Makin J, Pattinson R. The effect of pre-eclampsia on retinal microvascular caliber at delivery and post-partum. Obstet Med. 2018;11:116–20. 30214476 10.1177/1753495X17745727 PMC6134356

[7] Rahman I, Saleemi G, Semple D, Stanga P. Pre-eclampsia resulting in central retinal vein occlusion. Eye Lond Engl. 2006;20:955–7. 10.1038/sj.eye.6702065 16082392

[8] Sánchez Zamora P, Mejía Arnaud RA, Castro S, R., Gómez Del Pulgar Vázquez, B., Correa Barrera JJ. Bilateral serous retinal detachment in a patient with atypical presentation of preeclampsia due to HELLP syndrome. Rev Esp Anestesiol Reanim. 2002;69:114–118. 10.1016/j.redare.2020.11.014 35177366

[9] Bos AM, van Loon AJ, Ameln JG. [Serous retinal detachment in preeclampsia]. Ned Tijdschr Geneeskd. 1999;143:2430–2. 10608978

[10] Lee CS, Choi EY, Lee M, Kim H, Chung H. Serous retinal detachment in preeclampsia and malignant hypertension. Eye. 2019;33:1707–14. 31089238 10.1038/s41433-019-0461-8 PMC7002678

[11] Çelik G, Eser A, Günay M, Yenerel NM. Bilateral vision loss after delivery in two cases: severe preeclampsia and HELLP syndrome. Turk J Ophthalmol. 2015;45:271–3. 27800247 10.4274/tjo.45722 PMC5082267

[12] Cheung CY, Biousse V, Keane PA, Schiffrin EL, Wong TY. Hypertensive eye disease. Nat Rev Dis Primer. 2022;8:1–18. 10.1038/s41572-022-00342-0 35273180

[13] Wong W, Gopal L, Yip CC. Hypertensive retinopathy and choroidopathy. CMAJ Can Med Assoc J J Assoc Medicale Can. 2020;192:E371. 10.1503/cmaj.191275 PMC7145371 32392526

[14] Saito M, et al. Increased choroidal blood flow and choroidal thickness in patients with hypertensive chorioretinopathy. Graefes Arch Clin Exp Ophthalmol Albrecht Von Graefes Arch Klin Exp Ophthalmol. 2020;258:233–40. 10.1007/s00417-019-04511-y 31724089

[15] Mulè G, et al. Association between early-stage chronic kidney disease and reduced choroidal thickness in essential hypertensive patients. Hypertens Res Off J Jpn Soc Hypertens. 2019;42:990–1000. 10.1038/s41440-018-0195-1 30631159

[16] Geraci G, et al. Choroidal thickness is associated with renal hemodynamics in essential hypertension. J Clin Hypertens Greenwich Conn. 2020;22:245–53. 10.1111/jch.13777 PMC8030072 31945274

[17] Schiffrin EL, Park JB, Intengan HD, Touyz RM. Correction of arterial structure and endothelial dysfunction in human essential hypertension by the angiotensin receptor antagonist Losartan. Circulation. 2000;101:1653–9. 10758046 10.1161/01.cir.101.14.1653

[18] İçel E, İmamoğlu Hİ, Türk A, İçel A, Akyol N. A comparison of the effects of Perindopril arginine and amlodipine on choroidal thickness in patients with primary hypertension. Turk J Med Sci. 2018;48:1247–54. 30541254 10.3906/sag-1803-171

[19] Zhang Y, Zou J, Wan F, Peng F, Peng C. Update on the sources, pharmacokinetics, Pharmacological action, and clinical application of anisodamine. Biomed Pharmacother. 2023;161:114522. 37002581 10.1016/j.biopha.2023.114522

[20] Evans JR. Ginkgo biloba extract for age-related macular degeneration. Cochrane Database Syst Rev CD001775 2013. 10.1002/14651858.CD001775 10796819

[21] Zhang Y, et al. Using corneal confocal microscopy to compare mecobalamin intramuscular injections vs oral tablets in treating diabetic peripheral neuropathy: a RCT. Sci Rep. 2021;11:14697. 34282267 10.1038/s41598-021-94284-4 PMC8290034

[22] Yao H, et al. Comparison of the effects of prophylactic and therapeutic administrations on peripheral neuropathy in streptozotocin-diabetic rats with Gliclazide or methylcobalamin. Exp Clin Endocrinol Diabetes Off J Ger Soc Endocrinol Ger Diabetes Assoc. 2020;128:635–43. 10.1055/a-0635-0672 30453342

